# Nutrition and growth in preterm babies – are we measuring the right thing?

**DOI:** 10.1038/s41390-025-04270-z

**Published:** 2025-07-11

**Authors:** Frank Bloomfield, Tanith Alexander, Barbara Cormack

**Affiliations:** 1 Liggins Institute, University of Auckland, Private Bag 92019, Auckland, New Zealand; 2 Te Whatu Ora Health New Zealand Counties Manukau, Middlemore Hospital, Auckland, New Zealand; 3 Te Whatu Ora Health New Zealand Te Toka Tumai Auckland, Auckland City Hospital, Auckland, New Zealand

The systematic review and meta-analysis by Sanchez-Holgado et al.^1^ assesses the impact of the amount of enteral protein intake on growth, measured as incremental change in weight, length and head circumference. The novel features of this systematic review are that only studies that are randomised controlled trials in which babies received at least 50% of their enteral intake from fortified human milk are included and actual protein intakes had to be reported in at least one of the groups. Whilst other meta-analyses have focused on “high versus low” protein, this review utilised meta-regression to examine dose-response relationship between protein intake (g.Kg^−1^.d^−1^) and growth. It therefore addresses the question of the quantum of enteral protein intake in babies already receiving supplementary protein intake on growth outcomes.

The key finding is that each additional gram of protein per Kg bodyweight per day results in a mean (95% confidence intervals) increase in weight gain of 5.7 (2.3, 9.2) g.Kg^−1^.d^−1^, increasing to 8.8 (4.4, 13.2) g.Kg^−1^.d^−1^ after adjusting for energy intake. The Cochrane review of fortification versus no fortification reported a mean difference of only 3.8 (2.9, 4.7) g.Kg^−1^.d^−1^ with fortification^2^ and full fortification at a feed volume of 150 mL.Kg^−1^.d^−1^ provides approximately 1.0–2.2 g.Kg^−1^.d^−1^ additional protein.

The effect size reported by Sanchez-Holgado et al. for each additional g of protein per Kg body weight per day therefore seems high, although the confidence intervals are wide reflecting both the small sample sizes (16–77 per arm) and low quality of evidence, with the larger trials at high risk of bias. Although actual protein intakes were measured in at least one group for each included trial, with mean or median protein intakes 2.9 to 4.7 g.Kg^−1^.d^−1^, several types of milk analysers were used at varying time points across trials, introducing variability in protein measurement. Differences of up to 1 g.100 mL^−1^ may affect reported intakes,^3^ especially if based on crude rather than total protein, as this can overestimate bioavailable protein. Growth velocity is a calculated variable that does not account for sex, gestational age or birth centile and can be calculated using a variety of methods which can lead to very different results.^4^

Sanchez-Holgado et al.^1^ found minimal effect of protein on length gain, which was only statistically significant in the multivariate analysis adjusted for concurrent energy intake with a small effect size (0.8 [0.4, 1.2] mm per week; note this is absolute length gain, not proportional to current length) and a very small number of participants (total *n* = 174). Energy intake was negatively associated with linear growth (−0.26 [−0.47, −0.05] mm per week). In the Cochrane review of fortifiers with different protein content,^5^ the subgroup analyses of energy content found that in trials in which the fortifiers had similar energy content, higher protein intake increased length growth whereas in the trials that did not compare isocaloric fortifiers, this was not the case, supporting the suggestion by Sanchez-Holgado and colleagues that energy to protein ratios may require more of our attention.

What should we make of the findings in this review? The simple conclusion is that more enteral protein improves weight gain; however, it does not improve length gain, usually considered the real measure of growth whereas weight is a measure of mass. There was also no benefit for growth in head circumference; head circumference is correlated with brain volume^6^ which, in turn, is correlated with two-year neurodevelopmental outcomes.^7^ The manuscript draws conclusions about the protein intake likely required to match fetal growth (weight gain) rate, but how do we know that this is what we should be targeting?

The emphasis in the literature of reporting short-term outcomes, mostly weight gain, risks conflating weight gain with growth, incremental growth rate with the outcome of importance and, therefore, a target ‘growth rate’. We can be confident that ‘adequate’ nutrition is necessary for healthy development but is it time to consider how we should determine when nutrition is adequate or not? We suggest that incremental weight gain is not the right measure. Given how challenging it can be to measure length accurately in preterm babies, and also how head circumference can be distorted by both positional plagiocephaly and methods of securing CPAP, weight is both a convenient and relatively straightforward proxy for growth. However, some variation in growth is expected, and growth within a range likely is adequate to support healthy development. There has been increasing debate in recent years about the definition of ‘faltering growth’ and similar terms.^8^ This debate highlights the need to identify the in-hospital measure of growth that truly matters: when a baby’s growth trajectory is one that places the baby at risk of adverse long-term health outcomes, principally neurodevelopmental but conceivably also cardiometabolic. It would be interesting to know how many babies in the meta-analysis reported in the paper by Sanchez-Holgado et al. suffered faltering postnatal growth defined, for example, by a greater than 0.8 z-score decline in weight.^9^ Given the small sample sizes and the fact that these were trials of babies receiving at least 50% fortified breastmilk, one assumes very few, raising the question of whether, even if the large effect size reported is real, it really matters.

The outcome of real interest is often neurodevelopmental outcome, but we have to acknowledge that it will not always be possible, and for some trial hypotheses not appropriate, for trials to measure this outcome. Neither the Cochrane review on fortification^2^ nor a recent meta-analysis of higher versus lower protein intakes^10^ was able to report convincingly on long-term outcomes, because of the paucity and quality of data. Ideally, trials will become larger and focus on relevant long-term primary outcomes but, in the meantime, the neonatal nutrition research community would benefit from agreeing on the in-hospital growth outcome that is most relevant, how to define this and then how to measure and report this, which in itself will enhance meta-analysis. If this moves the outcome from a continuous variable to a dichotomous variable, the result will be an increase in the sample size of trials giving us greater confidence in the outcome. Ideally, this will be accompanied by including longer-term outcomes, such as two-year neurodevelopmental outcomes (acknowledging the limitations of this time point) that will support refinement of the definition of inadequate growth.

This would enable us to move on from a discussion about the ‘optimal’ growth rate in terms of a certain number of grams per Kilogram per day to one that focuses on preventing an outcome that we can be confident is associated with risk of adverse outcome.

## References

[1] Sanchez-Holgado, M. et al. Systematic review and meta-analysis of enteral protein intake effects on growth in preterm infants. *Pediatr. Res.*10.1038/s41390-025-04115-9 (2025). 40473805 10.1038/s41390-025-04115-9 PMC12602309

[2] Amissah, E. A., Brown, J. & Harding, J. E. Protein supplementation of human milk for promoting growth in preterm infants. *Cochrane Database Syst. Rev.* **9**, CD000433 (2020). 32964431 10.1002/14651858.CD000433.pub3 PMC8094919

[3] Kwan, C., Fusch, G., Rochow, N., Fusch, C. & Collaborators, M. S. Milk analysis using milk analyzers in a standardized setting (Mamas) study: a multicentre quality initiative. *Clin. Nutr.* **39**, 2121–2128 (2020). 31526612 10.1016/j.clnu.2019.08.028

[4] Fenton, T. R. et al. An attempt to standardize the calculation of growth velocity of preterm infants-evaluation of practical bedside methods. *J. Pediatr.* **196**, 77–83 (2018). 29246464 10.1016/j.jpeds.2017.10.005

[5] Gao, C., Miller, J., Collins, C. T. & Rumbold, A. R. Comparison of different protein concentrations of human milk fortifier for promoting growth and neurological development in preterm infants. *Cochrane Database Syst. Rev.* **11**, CD007090 (2020). 33215474 10.1002/14651858.CD007090.pub2 PMC8092673

[6] Cheong, J. L. et al. Head growth in preterm infants: correlation with magnetic resonance imaging and neurodevelopmental outcome. *Pediatrics* **121**, e1534–e1540 (2008). 18519457 10.1542/peds.2007-2671

[7] Lind, A. et al. Associations between regional brain volumes at term-equivalent age and development at 2 years of age in preterm children. *Pediatr. Radiol.* **41**, 953–961 (2011). 21534004 10.1007/s00247-011-2071-x

[8] Fenton, T. R. et al. Extrauterine growth restriction” and “postnatal growth failure” are misnomers for preterm infants. *J. Perinatol.* **40**, 704–714 (2020). 32214217 10.1038/s41372-020-0658-5

[9] Goldberg, D. L. et al. Identifying malnutrition in preterm and neonatal populations: recommended indicators. *J. Acad. Nutr. Diet.* **118**, 1571–1582 (2018). 29398569 10.1016/j.jand.2017.10.006

[10] Das, S. et al. High protein intake on later outcomes in preterm children: a systematic review and meta-analysis. *Pediatr. Res.* **97**, 67–80 (2025). 38858504 10.1038/s41390-024-03296-z PMC11798874

